# Requirement of digestible calcium at different dietary concentrations of digestible phosphorus for broiler chickens. 1. Broiler starters (d 1 to 10 post-hatch)

**DOI:** 10.1016/j.psj.2021.101439

**Published:** 2021-08-19

**Authors:** L.S. David, M.R. Abdollahi, M.R. Bedford, V. Ravindran

**Affiliations:** ⁎ Monogastric Research Centre, School of Agriculture and Environment, Massey University, Palmerston North 4442, New Zealand; † AB Vista, Marlborough, Wiltshire SN8 4AN, UK

**Keywords:** broiler, digestible calcium, digestible phosphorous, growth, bone

## Abstract

An experiment was conducted to determine the digestible calcium (**Ca**) and digestible phosphorous (**P**) requirements of 10-day-old broiler chickens. Fifteen corn-soybean meal-based diets containing 3.3, 3.9, 4.4, 5.0, and 5.5 g/kg standardized ileal digestible (**SID**) Ca and 4.0, 5.0, and 6.0 g/kg SID P was fed to broilers from d 1 to 10. Each experimental diet was randomly allocated to 6 replicate cages (12 birds per cage). Body weight and feed intake were recorded at the start and end of the experiment and the feed conversion ratio was calculated. On d 10, birds were euthanized to collect ileal digesta, toes and tibia for the determination of digestible Ca and P, toe ash concentration and the concentrations of ash, Ca, and P in tibia. Titanium dioxide (5 g/kg) was included in all diets as an indigestible indicator for apparent ileal digestibility measurements. Total excreta were collected from d 1 to 10 for the measurement of total tract retention of Ca and P. Fixed effects of the experiment were dietary concentrations of SID Ca and SID P and their interaction. If the interaction or main effects were significant (*P* < 0.05), the parameter estimates for second-order response surface model were determined using General Linear Model procedure of SAS software. The growth performance, bone mineralization and mineral utilization of broiler starters were found to be optimized at 5 g/kg SID P concentration. Required SID Ca for maximum weight gain and bone mineralization was determined to be 3.32 and 4.36 to 4.78 g/kg, respectively, at 5 g/kg SID P concentration, which correspond to SID Ca to SID P ratios of 0.66 and 0.87 to 0.96, respectively. The estimated SID Ca requirement for weight gain is lower than the current Ca recommendation (9.6 g/kg total Ca or 4.4 g/kg SID Ca) for broiler starters. However, bone mineralization is maximized around the current total Ca recommendation at 8.9 to 9.8 g/kg (4.36–4.78 g/kg SID Ca) and indicates that bone mineralization requires more Ca than growth performance.

## INTRODUCTION

Calcium is important to build up skeletal health and a wide range of functions in the body such as blood clotting, muscle contraction, nerve impulse transmission, egg production, enzyme activation, metabolic reactions, protein synthesis, maintenance of osmotic and acid-base balances, and components in membranes (Crenshaw, 2001). Dietary P also plays a vital role in skeletal development and synthesis of other tissues in broilers (Bertram, 1995). A balance needs to be maintained between Ca and P because of their close interaction which influences the absorption and utilization of both minerals (Crenshaw, 2001). In general, total Ca to available P ratio of 2:1 is being maintained in commercial broiler diets (Angel, 2013). Excess or deficiency in one of the minerals can lead to reduced utilization of the other (Shafey et al., 1991).

In recent years, digestible P has been suggested as preferred term to express P availability in feed ingredients (WPSA, 2013). However, in poultry, the requirement of P is still being considered on available P basis (Ross, 2019). In pigs, however, recommendations for the requirement of digestible Ca and digestible P for growth performance, bone ash and Ca retention have now been established (González-Vega et al., 2016a,b; Merriman et al., 2017; Lagos et al., 2019a, 2019b). These requirements are based on standardized total tract digestible (**STTD**) Ca and STTD P values and it was found that the requirement of STTD Ca was greater when bone ash was used as a response criterion compared to growth performance. Similar comprehensive studies in poultry are non-existent and total Ca still being used in feed formulations. The shift to digestible P system may result in the oversupply of Ca. Unlike pigs, the nutrient digestibility measured at the terminal ileum (Lemme et al., 2004) is unquestionably accepted in poultry because of limitations in the total tract digestibility measurement (Ravindran et al., 1999). There is only one report available on the digestible Ca requirement of broilers (Angel, 2018), but no experimental data were provided. The requirements of digestible Ca and digestible P for 0 to 10 d old broilers were proposed to be 6.1 and 5.3 g/kg, respectively, which correspond to a digestible Ca to digestible P ratio of 1.15 (Angel, 2018; van Krimpen et al., 2016). Clearly research is warranted to investigate the requirements of digestible Ca and digestible P for broilers. Toward this end, the current study was designed to determine the requirements of standardized ileal digestible (**SID**) Ca and SID P for broiler starters (d 1–10 post-hatch) with different concentrations of digestible Ca and digestible P. Because ileal Ca digestibility coefficients have been reported recently for the main Ca sources in broilers (Anwar, 2015, 2016a, 2016b, 2016c, 2017, 2018; David et al*.,* 2019, 2020a,b, 2021) and it is now possible to formulate diets based on the digestible Ca in feed ingredients. The objective of the current study was to determine the requirements for digestible Ca and digestible P in broiler chickens during d 1 to 10 post-hatch to maximize growth performance, bone mineralization and Ca and P retention.

## MATERIALS AND METHODS

The experiment was conducted according to the New Zealand Revised Code of Ethical Conduct for the use of live animals for research, testing and teaching, and approved by the Massey University Animal Ethics Committee.

### Experimental Diets

The ingredients (corn, soybean meal, limestone, dicalcium phosphate, and monosodium phosphate) were obtained from commercial sources and analyzed for nutrient composition. The analyzed Ca and P concentrations were used to formulate the assay diets.

The recommended requirements of total Ca and available P for Ross 308 broiler starters (1–10 d post-hatch) are 9.6 and 4.8 g/kg, respectively (Ross, 2019). Based on published values of digestible Ca and P in feed ingredients (Tables 1 and 2), equivalent SID Ca and SID P values were 4.4 and 5.4 g/kg, respectively. Therefore, a range of digestible Ca (3.3–5.5 g/kg) and digestible P (4–6 g/kg) which are below and above these recommended values were considered in the development of treatments. Fifteen experimental starter diets based on corn-soybean meal were formulated in a 5 × 3 factorial arrangement with diets containing five concentrations of Ca and 3 concentrations of P (Table 3). Diets were formulated to contain 3.3, 3.9, 4.4, 5.0, and 5.5 g/kg SID Ca (corresponding to 7, 8, 9, 10, and 11 g/kg total Ca, respectively), and 4, 5, and 6 g/kg SID P (corresponding to 5.3, 6.8, and 8.3 g/kg total P, respectively) as indicated in Table 4. Concentrations of SID Ca ranged from 0.73 to 1.15 times the requirement for total Ca (Ross, 2019). All experimental diets were isoenergetic and isonitrogenous, and formulated by varying the inclusions of corn, soybean meal, and soybean oil. Each diet was separately mixed and pelleted. The diets were steam-conditioned to 70°C for 30 s and pelleted using a pellet mill (Model Orbit 15; Richard Sizer Ltd., Kingston-upon-Hull, UK) capable of manufacturing 180 kg of feed/h and equipped with a die ring with 3 mm holes and 35 mm thickness.

**Table 1:** Total and standardized ileal digestible (SID) phosphorous (P) contents of feed ingredients.

Ingredient	Total P (g/kg)^1^	SID P digestibility (%)	SID P (g/kg)
Corn	2.30	70^2^	1.61
Soybean meal	5.90	75^2^	4.43
Dicalcium phosphate	185	79^3^	146
Monosodium phosphate	225	67^4^	151

1 Analyzed values.

2 Mutucumarana et al. (2015).

3 van Harn et al. (2017).

4 Shastak et al. (2012).

**Table 2:** Total and standardized ileal digestible (SID) calcium (Ca) content of feed ingredients.

Ingredients	Total Ca (g/kg)^1^	SID Ca digestibility (%)	SID Ca (g/kg)
Corn	0.20	50^2^	0.10
Soybean meal	3.50	54^3^	1.89
Dicalcium phosphate	260	36^4^	93.6
Limestone	410	55^4^^,^^5^	226

1 Analyzed values.

2 Assumed value.

3 David et al. (2020b).

4 David et al. (2019).

5 Anwar et al. (2016c).

**Table 3:** Ingredient composition of experimental diets (g/kg, as fed basis).

SID Ca	3.3			3.9			4.4			5.0			5.5		
SID P	4.0	5.0	6.0	4.0	5.0	6.0	4.0	5.0	6.0	4.0	5.0	6.0	4.0	5.0	6.0
Total calcium	7.0	7.0	7.0	8.0	8.0	8.0	9.0	9.0	9.0	10.0	10.0	10.0	11.0	11.0	11.0
SID Ca: SID P	0.84	0.67	0.56	0.97	0.78	0.65	1.11	0.89	0.74	1.25	1.00	0.83	1.39	1.11	0.92
Corn	568	565	553	563	560	548	558	555	543	553	550	538	548	545	533
Soybean meal	361	362	364	362	362	364	363	363	365	363	364	366	364	365	367
Dicalcium phosphate	10.1	10.1	10.1	10.1	10.1	10.1	10.1	10.1	10.1	10.2	10.2	10.2	10.2	10.2	10.2
Monosodium phosphate	0.00	6.7	13.4	0.00	6.7	13.4	0.00	6.7	13.4	0.00	6.7	13.4	0.00	6.7	13.4
Limestone	7.3	7.3	7.3	9.7	9.7	9.7	12.2	12.2	12.2	14.6	14.6	14.6	17.0	17.0	17.0
Sodium chloride	0.7	0.7	0.7	0.7	0.7	0.7	0.7	0.7	0.7	0.7	0.7	0.7	0.7	0.7	0.7
Sodium bicarbonate	5.9	1.0	0.00	5.9	1.0	0.00	5.9	1.0	0.00	5.8	1.0	0.00	5.8	1.0	0.00
DL Methionine	3.8	3.8	3.8	3.8	3.8	3.8	3.8	3.8	3.8	3.8	3.8	3.8	3.8	3.8	3.8
Lysine HCl	4.8	4.8	4.8	4.8	4.8	4.8	4.8	4.8	4.8	4.8	4.8	4.7	4.8	4.8	4.7
L Threonine	2.7	2.7	2.7	2.7	2.7	2.7	2.7	2.7	2.7	2.7	2.7	2.7	2.7	2.7	2.7
L Valine	1.3	1.3	1.3	1.3	1.3	1.3	1.3	1.3	1.3	1.3	1.3	1.3	1.3	1.3	1.3
Vitamin premix^1^,^2^	1.0	1.0	1.0	1.0	1.0	1.0	1.0	1.0	1.0	1.0	1.0	1.0	1.0	1.0	1.0
Trace mineral premix^2^	1.0	1.0	1.0	1.0	1.0	1.0	1.0	1.0	1.0	1.0	1.0	1.0	1.0	1.0	1.0
Choline chloride 60%	0.8	0.8	0.8	0.8	0.8	0.8	0.8	0.8	0.8	0.8	0.8	0.8	0.8	0.8	0.8
Titanium dioxide	5.0	5.0	5.0	5.0	5.0	5.0	5.0	5.0	5.0	5.0	5.0	5.0	5.0	5.0	5.0
Soybean oil	26.4	27.7	31.7	28.1	29.4	33.4	29.8	31.1	35.1	31.5	32.8	36.8	33.2	34.5	38.5

Abbreviations: SID Ca, standardized ileal digestible calcium; SID P, standardized ileal digestible phosphorous.

1 Supplied per kilogram of diet: vitamin A (trans-retinyl acetate), 12,000 IU; cholecalciferol, 4,000 IU; thiamine, 3 mg; riboflavin, 9 mg; pyridoxine, 10 mg; folic acid, 3 mg; biotin, 0.25 mg; cyanocobalamin, 0.02 mg; dl-α-tocopherol acetate, 80 IU; niacin, 60 mg; Ca-D pentothenate, 15 mg; menadione, 4 mg; choline chloride, 600 mg; Co, 0.25 mg; I, 1.5 mg; Mo, 0.25 mg; Se, 0.26 mg; Mn, 100 mg; Cu, 10 mg; Zn, 80 mg; Fe, 60 mg; antioxidant, 100 mg.

2 Vitamin and trace mineral premixes contained no calcium.

**Table 4:** Calculated and analyzed nutrient composition of experimental diets (g/kg, as fed basis).

SID Ca	3.3			3.9			4.4			5.0			5.5		
SID P	4.0	5.0	6.0	4.0	5.0	6.0	4.0	5.0	6.0	4.0	5.0	6.0	4.0	5.0	6.0
SID Ca: SID P	0.84	0.67	0.56	0.97	0.78	0.65	1.11	0.89	0.74	1.25	1.00	0.83	1.39	1.11	0.92
Total Ca	7.0	7.0	7.0	8.0	8.0	8.0	9.0	9.0	9.0	10.0	10.0	10.0	11.0	11.0	11.0
Non-phytate P	3.37	4.87	6.38	3.37	4.87	6.39	3.38	4.88	6.39	3.38	4.88	6.40	3.39	4.89	6.40
Total Ca: Non-phytate P	2.08	1.44	1.10	2.37	1.64	1.25	2.66	1.85	1.41	2.96	2.05	1.56	3.25	2.25	1.72
Dry matter	883	882	878	882	881	878	880	879	876	879	878	875	878	877	873
AME (kcal/kg)	3,000	3,000	3,000	3,000	3,000	3,000	3,000	3,000	3,000	3,000	3,000	3,000	3,000	3,000	3,000
Crude protein	220	220	220	220	220	220	220	220	220	220	220	220	220	220	220
Digestible protein	179	179	179	179	179	179	179	179	179	179	179	179	179	179	179
Starch	356	354	347	353	351	344	350	348	340	347	345	337	344	342	334
Crude fat	47.9	48.9	52.2	49.3	50.3	53.6	50.7	51.7	55.0	52.1	53.1	56.4	53.5	54.5	57.8
Crude fiber	28.3	28.2	28.1	28.2	28.2	28.0	28.2	28.1	27.9	28.1	28.0	27.9	28.0	28.0	27.8
Total Ca	7.0	7.0	7.0	8.0	8.0	8.0	9.0	9.0	9.0	10.0	10.0	10.0	11.0	11.0	11.0
SID Ca	3.33	3.33	3.33	3.88	3.88	3.88	4.43	4.43	4.43	4.98	4.98	4.98	5.53	5.53	5.53
Total P	5.30	6.80	8.30	5.30	6.80	8.30	5.30	6.80	8.30	5.30	6.80	8.30	5.30	6.80	8.30
Phytate P	1.93	1.93	1.92	1.93	1.93	1.91	1.92	1.92	1.91	1.92	1.92	1.90	1.91	1.91	1.90
SID P	4.0	5.0	6.0	4.0	5.0	6.0	4.0	5.0	6.0	4.0	5.0	6.0	4.0	5.0	6.0
Chloride	1.9	1.9	1.9	1.9	1.9	1.9	1.9	1.9	1.9	1.9	1.9	1.9	1.9	1.9	1.9
Sodium	2.3	2.3	3.3	2.3	2.3	3.3	2.3	2.3	3.3	2.3	2.3	3.3	2.3	2.3	3.3
Potassium	11	11	11	11	11	11	11	11	11	11	11	11	11	11	11
Choline (mg/kg)	1,700	1,700	1,700	1,700	1,700	1,700	1,700	1,700	1,700	1,700	1,700	1,700	1,700	1,700	1,700
Dig. threonine	8.60	8.60	8.60	8.60	8.60	8.60	8.60	8.60	8.60	8.60	8.60	8.60	8.60	8.60	8.60
Dig. alanine	8.13	8.12	8.09	8.12	8.11	8.08	8.10	8.10	8.07	8.09	8.08	8.06	8.08	8.07	8.05
Dig. valine	9.60	9.60	9.60	9.60	9.60	9.60	9.60	9.60	9.60	9.60	9.60	9.60	9.60	9.60	9.60
Dig. isoleucine	7.29	7.29	7.30	7.30	7.30	7.30	7.30	7.30	7.31	7.30	7.30	7.31	7.30	7.30	7.31
Dig. leucine	15.0	15.0	15.0	15.0	15.0	15.0	15.0	15.0	14.9	15.0	15.0	14.9	15.0	14.9	14.9
Dig. lysine	12.8	12.8	12.8	12.8	12.8	12.8	12.8	12.8	12.8	12.8	12.8	12.8	12.8	12.8	12.8
Dig. arginine	12.6	12.6	12.6	12.6	12.6	12.6	12.6	12.6	12.6	12.6	12.6	12.6	12.6	12.6	12.6
Dig. cysteine	2.93	2.93	2.92	2.93	2.93	2.91	2.92	2.92	2.91	2.92	2.92	2.90	2.91	2.91	2.90
Dig. methionine	6.57	6.57	6.58	6.57	6.57	6.59	6.58	6.58	6.59	6.58	6.58	6.60	6.59	6.59	6.60
Dig. methionine + cysteine	9.50	9.50	9.50	9.50	9.50	9.50	9.50	9.50	9.50	9.50	9.50	9.50	9.50	9.50	9.50
Analyzed values^1^															
Dry matter	883	882	878	882	881	878	880	879	876	879	878	875	878	877	873
Total Ca	6.9	7.1	7.3	7.9	7.7	7.8	8.2	9.2	9.1	11.2	10.7	10.2	11.4	11.6	11.0
Total P	5.5	7.5	8.7	5.6	7.1	8.6	6.9	7.4	9.0	6.8	7.5	8.7	5.9	7.6	8.9

Abbreviations: AME, apparent metabolisable energy; Ca, calcium; Dig., digestible; P, phosphorous; SID, standardized ileal digestible.

1 Samples were analyzed in triplicate.

### Birds

A total of 1,080, day-old male broilers (Ross 308) were obtained from a commercial hatchery, weighed, and allocated (mean ± SD, 45 ± 0.41 g) to 90 electrically heated battery brooders (12 per brooder cage). Each of the 15 diets was randomly assigned into 6 replicate cages. The experimental diets were offered ad libitum from d 1 to 10 post-hatch and water was available at all the times.

### Measurements

#### Growth Performance

Body weights and feed intake were recorded on a cage basis at the start and end of the experimental period. Mortality was recorded daily. Feed conversion ratio (**FCR**) was corrected for the body weight of any bird that died during the experiment.

#### Ileal Digestibility and Apparent Total Tract Retention of Ca and P

At the end of the experiment, 8 birds per replicate were euthanized by cervical dislocation and the contents of the lower half of ileum was collected by flushing the contents gently with distilled water into plastic containers and processed as described by Ravindran et al. (2005). Total excreta samples were collected during all 10 d, pooled within a cage and processed as described by David et al. (2019).

#### Bone Mineralization

Middle toe of the left feet and right tibia were removed from 8 birds per replicate (from the birds euthanized for ileal digesta collection) and immediately frozen at −20°C. Tibiae were cleaned from all adherent tissues and were kept frozen in airtight plastic bags until the measurements. Tibiae were oven dried at 105°C for 24 h, de-fatted by refluxing petroleum ether in a Soxhlet apparatus for 16 h, oven-dried at 105°C overnight for dry defatted bone weight determination and ashed in ceramic crucibles for 24 h at 600°C for fat-free ash weight determination. Tibia ash content was expressed as a percentage of dry bone weight. The toe samples were weighed and dry ashed at 550°C for 24 h for determination of toe ash (Ravindran et al., 1995). Tibia Ca and P concentrations were determined and expressed as g/kg dried defatted bone.

#### Carcass Retention of Ca and P

At the start of the trial (d 1), 10 additional chicks were killed by cervical dislocation. At the end of experiment (d 10), 4 birds per replicate were randomly selected, fasted overnight, weighed and killed by cervical dislocation with minimum blood loss. At both ages, feathers were removed, the carcass weight was recorded and defeathered carcasses were stored at −20°C. In this paper, the term “carcass” refers to the whole body without feathers. The frozen carcasses were cut into small pieces and minced twice to obtain homogenous subsamples.

### Chemical Analysis

Ingredients were analyzed for dry matter (**DM**, method 930.15; AOAC, 2016), ash (method 942.05; AOAC, 2016), nitrogen (968.06; AOAC, 2016), fat (AOAC 2003.06), crude fiber (AOAC 2002.04), Ca and total P (method 968.08D; AOAC, 2016), and phytate P (Caldwell, 1992). The concentrations of ash (AOAC 942.05; AOAC, 2016), Ca and P (AOAC 968.08D; AOAC, 2016) of tibia and the concentration of toe ash (AOAC 942.05; AOAC, 2016) were determined using standard procedures. The diet, ileal digesta, and excreta samples were analyzed for DM (method 930.15; AOAC, 2016), Ca (Karlsson et al., 2014) total P (method 968.08D; AOAC, 2016), and titanium dioxide (Short et al., 1996). Subsamples of the minced carcass were analyzed for DM (method 930.15; AOAC, 2016), Ca, and P (AOAC 968.08D; AOAC, 2016).

### Calculations

The apparent ileal digestibility (**AID**) coefficients of Ca and P were calculated using titanium marker ratios in the diet and ileal digesta (Ravindran et al., 1999) as indicated below. Analyzed values were used in digestibility and retention calculations.

$$\text{AID}\;\text{coefficient}\;\text{of}\;\text{Ca}\;\text{or}\;P\;=\;1-\left[\left(Ti_{I}/Ti_{O}\right)\;\times \;\left(M_{O}/M_{I}\right)\right]$$

where Ti_I_ is the titanium concentration in the diet, Ti_O_ is the titanium concentration in the ileal digesta, *M*_O_ is the concentration of Ca or P in the ileal digesta, and *M*_I_ is the concentration of Ca or P in the diet. All concentrations were expressed as g/kg DM.

Standardized ileal digestibility (**SID**) coefficients of Ca and P were then calculated, based on previously determined values for endogenous Ca (108 mg/kg DM intake, Anwar, 2016) and P (25 mg/kg DM intake, Mutucumarana and Ravindran, 2020) values, as follows:

$$\text{SID}\;=\;\text{AID}\;+\;\left(IEL/M_{I}\right)$$

where SID and AID represent the coefficients of standardized ileal digestibility and AID of Ca or P, respectively, IEL represents the ileal endogenous losses (mg/kg DM intake) of Ca or P and *M*_I_ represents the concentration of Ca or P in the diet (g/kg DM).

The apparent total tract retention (**ATTR**) coefficient of Ca and P (% intake) was calculated using following equation:

$$\text{ATTR}\;\text{coefficient}\;\text{of}\;\text{Ca}\;\text{or}\;P\;=\;\left[\left(M_{I}\;\times \;FI\right)\;-\;\left(M_{E}\;\times \;EO\right)/M_{I}\;\times \;FI\right]$$

where *M*_I_ is the concentration of Ca or P in the diet (g/kg DM), FI is the feed intake of birds (g, DM basis), *M*_E_ is the concentration of Ca or P in the excreta (g/kg DM) and EO is the excreta output (g, DM basis).

The intake of SID Ca or P and the retained Ca or P (g/bird) were calculated using following equations:

$$\text{Intake}\;\text{of}\;\text{SID}\;\text{Ca}\;\text{or}\;P\;=\;\left(FI\;\times \;M_{I}\;\times \;SID\;coefficient\right)$$

$$\text{Retained}\;\text{Ca}\;\text{or}\;P\;=\;\left(FI\;\times \;M_{I}\;\times \;ATTR\;coefficient\right)$$

where FI is the feed intake of birds (g/bird, DM basis), *M*_I_ is the concentration of Ca or P in the diet (g/kg DM).

The retained Ca or P (g/bird) in the carcass was calculated using following equation:

$$\text{Retained}\;\text{Ca}\;\text{or}\;P\;=\;\left[{\left(\text{Mc}\;\times \;\text{CW}\right)}_{D10}\;-\;{\left(\text{Mc}\;\times \;\text{CW}\right)}_{D0}\right]$$

where *M*_c_ is the concentration of Ca or P in the carcass (g per kg DM), CW is the carcass weight (g/bird) and D10 and D0 denote 10-day-old and day-old birds, respectively.

### Statistical Analysis

Data were analyzed using the General Linear Model (**GLM**) procedure of SAS (2019), with cage serving as the experimental unit. Two sets of analyses were conducted. First, as a factorial arrangement of treatments examining the effects of dietary concentrations of SID Ca and SID P and their interaction. The effects were considered significant at *P* ≤ 0.05. Second, if the interaction or main effects were significant, then the parameter estimates for the second-order response surface model were determined using GLM procedure of SAS (2019). All calculations started with the full model, but if needed, the model was reduced by removing parameter estimates that were not significant (*P* > 0.05) and the estimates were recalculated using the reduced model as described by González-Vega et al. (2016b). Linear and quadratic effects of both SID Ca and SID P and the interaction between SID Ca and SID P were included in the full model as follows:

$$Y\;=\;a\;+\;b\;\times \;\text{SID}\;\text{Ca}\;+\;c\;\times \;\text{SID}\;Ca^{2}\;+\;d\;\times \;\text{SID}\;P\;+\;e\;\times \;\text{SID}\;P^{2}\;+\;f\;\times \;\text{SID}\;\text{Ca}\;\times \;\text{SID}\;P$$

where Y is the dependent variable, a is the intercept, b, c, d, e, and f are the coefficients, and SID Ca and SID P are the concentrations (g/kg) of dietary SID Ca and SID P.

The concentrations of SID Ca at the maximum response values were calculated using the following equation:

$$SID\;Ca_{\max}\left(g/kg\right)\;=\;\left[\left(-f\;\times \;SID\;P\right)\;-b\right]\;/\;\left(2\;\times \;c\right)$$

where SID Ca_max_ is the concentration of SID Ca at the maximum response and SID P is the concentration of SID P in the diet.

The maximum response values were, therefore, calculated using the respective model equations with the concentrations of SID Ca at the maximum response for each concentration of SID P.

The relationships between measured parameters were analyzed by Pearson Correlations (SAS, 2019).

## RESULTS

Determined concentrations of SID Ca and SID P of the 15 assay diets, in comparison with formulated values, are summarized in Table 5. The calculated concentrations of SID Ca, based on published ingredient values (Table 2) were closer to those determined, except in 3 out of 15 diets. However, the differences were observed between the calculated and determined SID P values, especially at higher SID Ca concentrations.

### Growth Performance

**Figure 1:**
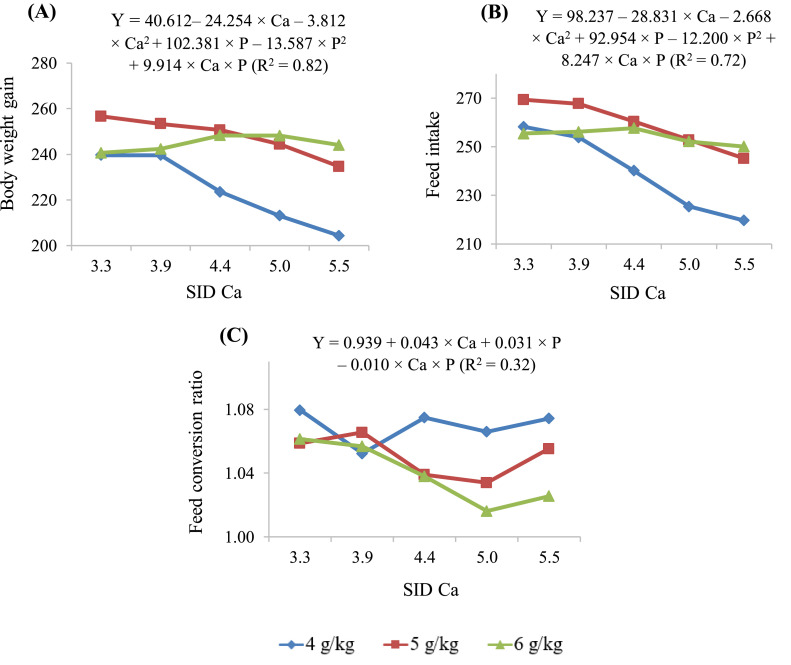
(A) Body weight gain (g/bird), (B) feed intake (g/bird) and (C) feed conversion ratio of broiler chickens fed different standardized ileal digestible (SID) calcium (Ca) and SID phosphorous (P) concentrations (4, 5, and 6 g/kg) from d 1 to 10.

The body weight gain, feed intake, and FCR from 0 to 10 d of chicks fed diets containing different concentrations of SID Ca and SID P are summarized in Table 6 and the trends are illustrated in Figure 1. There were interactions (*P* < 0.001) between SID Ca and SID P for all growth parameters. For the body weight gain and feed intake, the full model was used to predict the SID Ca at maximum response. However, a reduced model was used for FCR. At lower SID Ca concentrations (3.3 and 3.9 g/kg), the weight gain was higher at 5 g/kg SID P concentration than at 4 and 6 g/kg. However, the weight gain was higher at 6 g/kg SID P concentration if the dietary SID Ca was 5.5 g/kg and was lower at 4 g/kg SID P if the SID Ca concentrations were above 4.4 to 5.5 g/kg. At 4 g/kg SID P concentration, the weight gain was observed to be depressed (*P* < 0.001) with increasing SID Ca concentrations. The predicted maximum body weight gains at SID P concentrations of 4, 5, and 6 g/kg were 248, 255, and 247 g/bird, at SID Ca concentrations of 2.02, 3.32, and 4.62 g/kg, respectively. These values correspond to SID Ca to SID P ratios of 0.51, 0.66, and 0.77, respectively.

Similarly, feed intake was higher (*P* < 0.05) at 5 g/kg SID P if the SID Ca concentration was below 4.4 g/kg and was lower at 4 g/kg SID P if the SID Ca concentrations were above 4.4 g/kg. The predicted maximum feed intake at SID P concentrations of 5 and 6 g/kg were 272 and 257 g/bird, at SID Ca concentrations of 2.33 and 3.87 g/kg, respectively. These values correspond to SID Ca to SID P ratios of 0.47 and 0.65, respectively. Maximum value was not calculated for feed intake at 4 g/kg due to the lack of response. Maximum values were not calculated for FCR due to the linear nature of the response.

### Standardized Ileal Ca and P Digestibility Coefficients, Intake of Both SID Ca and SID P, and the Ratio between SID Ca and SID P Intakes

**Figure 2:**
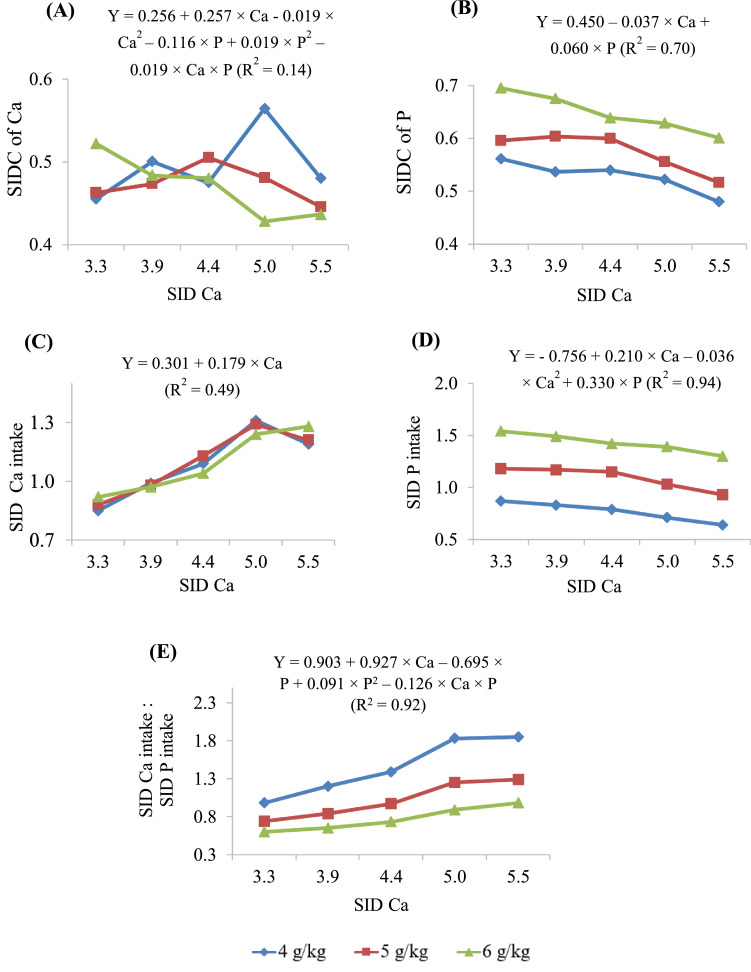
(A) Standardized ileal digestibility coefficients (SIDC) of calcium (Ca) and (B) phosphorous (P); intake (g/bird) of (C) standardized ileal digestible (SID) Ca and (D) SID P; and (E) ratio of SID Ca intake: SID P intake, of broiler chickens fed different concentrations of SID Ca and SID P (4, 5, and 6 g/kg) from d 1 to 10.

Data on standardized ileal digestibility coefficients of Ca and P, the intake of SID Ca and SID P and the ratio of SID Ca intake to SID P intake in 1 to 10 d old birds are presented in Table 7 and Figure 2. The reduced model was used to predict these parameters. For the standardized ileal Ca digestibility, the main effects and their interaction were not significant and therefore, the maximum values were not calculated. The digestible Ca intake was influenced by dietary SID Ca concentrations where the intake linearly increased (*P* < 0.001) as the SID Ca concentration increased. Therefore, the maximum values were not calculated for digestible Ca intake. Standardized ileal P digestibility linearly increased (*P* < 0.001) with increasing SID P and reducing SID Ca concentrations. The intake of digestible P was increased (*P* < 0.001) with increasing SID P concentration and with decreasing SID Ca concentrations. The predicted maximum intake of digestible P at SID P concentrations of 4, 5, and 6 g/kg were 0.80, 1.09, and 1.38 g/bird, at the SID Ca concentration of 2.91 g/kg. These values correspond to SID Ca to SID P ratios of 0.73, 0.58, and 0.49, respectively. The ratio of SID Ca intake to SID P intake was increased (*P* < 0.001) by increasing Ca concentrations and decreasing SID P concentrations, resulting a SID Ca × SID P interaction (*P* < 0.001). The maximum values were not calculated for the SID P intake and the ratio of SID Ca intake: SID P intake due to the linear Ca effect.

### Bone Mineralization

**Figure 3:**
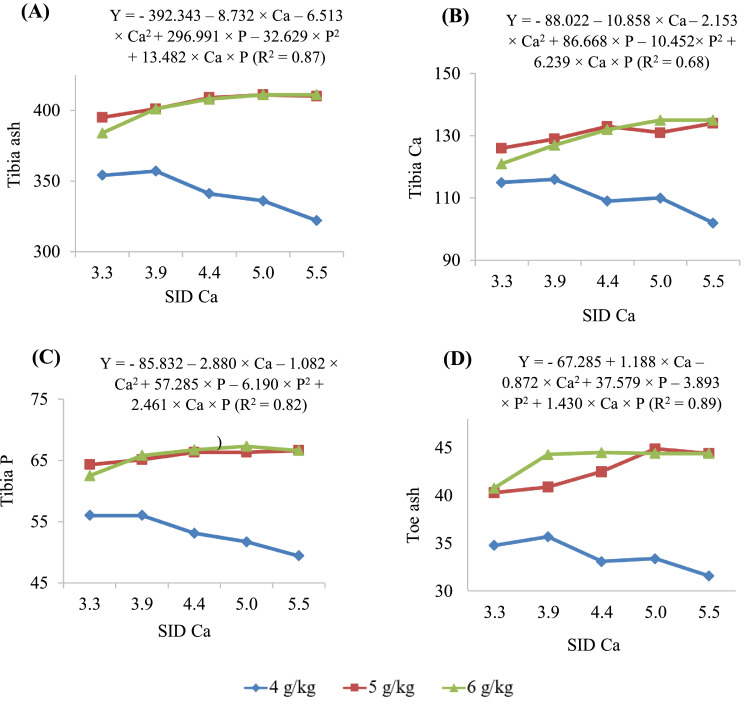
Concentrations (g/kg dried defatted matter) of (A) ash, (B) calcium (Ca) and (C) phosphorous (P) of tibia and (D) toe ash concentration (g/kg, as received basis) in broiler chickens fed different concentrations (g/kg) of standardized ileal digestible (SID) Ca and SID P (4, 5, and 6 g/kg) from d 0 to 10.

Table 8 and Figure 3 present the concentrations of ash, Ca and P of tibia, and toe ash content of 10-day-old broilers fed diets containing different SID Ca and SID P. The full model was used to predict the tibia parameters. The predicted maximum tibia ash concentration at SID P concentrations of 4, 5, and 6 g/kg was 352, 409, and 415 g/kg, at SID Ca concentrations of 3.47, 4.51, and 5.54 g/kg, respectively. These values correspond to SID Ca to SID P ratios of 0.87, 0.90, and 0.92, respectively. The predicted maximum tibia Ca content at SID P concentrations of 4, 5, and 6 g/kg was 115, 132, and 138 g/kg, at SID Ca concentrations of 3.27, 4.72, and 6.17 g/kg, respectively. These values correspond to SID Ca to SID P ratios of 0.82, 0.94, and 1.03, respectively. The predicted maximum tibia P content at SID P concentrations of 4, 5, and 6 g/kg was 55.5, 66.4, and 67.7 g/kg, at SID Ca concentrations of 3.22, 4.36, and 5.49 g/kg, respectively. These values correspond to SID Ca to SID P ratios of 0.80, 0.87, and 0.92, respectively. The toe ash was increased by increasing SID P concentrations at SID Ca concentrations of 3.3, 3.9, and 4.4 g/kg. The predicted maximum concentration of toe ash at SID P concentrations of 4, 5, and 6 g/kg was 34.4, 43.2, and 45.4 g/kg, and at SID Ca concentrations of 3.96, 4.78, and 5.60 g/kg, respectively. These values correspond to SID Ca to SID P ratios of 0.99, 0.96, and 0.93, respectively.

### Coefficients of ATTR and Retained Ca and P

Table 9 summarises the total tract retained Ca and P in 10-day-old birds fed diets containing different SID Ca and SID P. Apparent total tract retention coefficient (**ATTRC**) of Ca was lower at 4 g/kg SID P and increased with increasing SID P concentrations at all SID Ca concentrations. But the magnitude of increases was greater as the SID Ca concentrations increased, resulting in a SID Ca × SID P interaction (*P* < 0.05). A similar trend was observed for the total tract retained Ca (g/bird). The maximum values were not estimated for these parameters due to the linear Ca effect.

At all SID Ca concentrations, the ATTRC of P was reduced at 6 g/kg SID P, but the magnitude of reductions differed in different SID Ca. This resulted in a SID Ca × SID P interaction (*P* < 0.001). Retained P (g/bird), on the other hand, increased at or above 5 g/kg SID P and the degree of increment varied at different SID Ca concentrations, resulting in a SID Ca × SID P interaction (*P* < 0.001).

**Figure 4:**
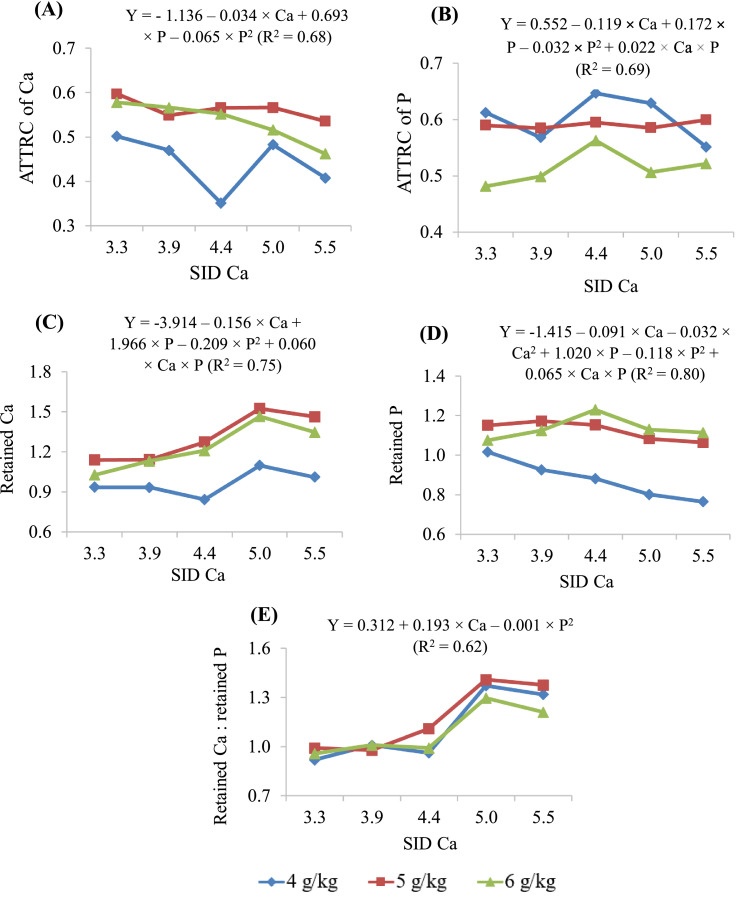
Apparent total tract retention coefficient (ATTRC) of (A) Ca and (B) phosphorous (P); retained (g/bird) (C) Ca and (D) P; (E) ratio of retained Ca to retained P, of broiler chickens fed different concentrations (g/kg) of standardized ileal digestible (SID) Ca and SID P (4, 5, and 6 g/kg) from d 0 to 10.

The predicted maximum retained P (g/bird) at SID P concentrations of 4, 5, and 6 g/kg was 1.00, 1.16, and 1.16 g/bird, at SID Ca concentrations of 2.62, 3.64, and 4.65 g/kg, respectively. These values correspond to SID Ca to SID P ratios of 0.66, 0.73, and 0.78, respectively. The ratio between retained Ca and retained P was higher (*P* < 0.05) at or above 5.0 g/kg SID Ca concentrations (Figure 4). The ratio was unaffected (*P* > 0.05) by SID P when the SID Ca was less than 4.4 g/kg and variable responses were noted at levels above 5.0 g/kg Ca, resulting in a SID Ca × SID P interaction (*P* < 0.05). Figure 4 illustrates the retention of Ca and P in 10-day-old broiler chickens fed different SID Ca and SID P.

### Carcass Retention of Ca and P

**Figure 5:**
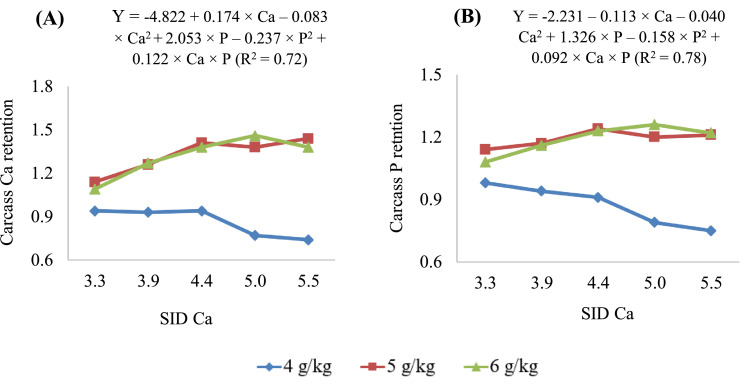
Carcass retention (g/bird) of (A) calcium (Ca), and (B) phosphorous (P) in 10-day-old broiler chickens fed different concentrations (g/kg) of standardized ileal digestible (SID) Ca and SID P (4, 5, and 6 g/kg).

Data on retention of Ca and P in the carcass of 10-day-old birds fed diets containing different SID Ca and SID P are presented in Table 10 and Figure 5. A reduced model was used for these parameters. An interaction (*P* < 0.05) was observed between SID Ca and SID P for carcass retention of Ca and P (Figure 5). At all SID Ca concentrations, the carcass Ca retention was increased at 5 and 6 g/kg SID P, but the magnitude of increment was greater with increasing SID Ca. The predicted maximum carcass Ca retention at SID P concentrations of 4, 5, and 6 g/kg was 0.93, 1.38, and 1.46 g/bird, at SID Ca concentrations of 4.00, 4.74, and 5.47 g/kg, respectively. These values correspond to SID Ca to SID P ratios of 1.00, 0.95, and 0.91, respectively. Similar to the carcass Ca retention, at all SID Ca concentrations, the carcass P retention was increased at 5 and 6 g/kg SID P, but the magnitude of increment was greater with increasing SID Ca. The predicted maximum carcass P retention at SID P concentrations of 4, 5, and 6 g/kg was 0.96, 1.22, and 1.26 g/bird, at SID Ca concentrations of 3.19, 4.35, and 5.50 g/kg, respectively. These values correspond to SID Ca to SID P ratios of 0.80, 0.87, and 0.92, respectively.

## DISCUSSION

In general, analyzed concentrations (Table 11) of proximate components, phytate, Ca, and P of ingredients (corn, soybean meal, limestone, dicalcium phosphate, and monosodium phosphate) were within the range reported in the literature (National Research Council 1994, National Research Council, 2012; Browning and Cowieson, 2014; Mutucumarana et al., 2014b, 2014c, 2015).

A noteworthy finding of the current work was that the calculated SID Ca concentrations of the 15 dietary treatments, in general, were representative of the determined SID concentrations. The closeness between determined and formulated values provides confidence in the published values for the SID Ca of ingredients. The similarity also provides indirect evidence for the additivity of SID Ca in ingredients when combined in feed mixtures. It must be noted that the SID Ca coefficients used in the calculation of dietary SID concentrations were determined in our laboratory and that the same Ca sources (limestone and dicalcium phosphate) evaluated in Ca digestibility assays were used in the formulation of treatment diets. On the other hand, calculated and determined SID P concentrations differed by as much as 25%. The SID P coefficients used in our calculations were from literature (Table 2) and the differences likely to reflect the variations reported among laboratories as demonstrated in a ring test involving 17 research stations by Rodehutscord et al. (2017).

### Requirements for SID P to Maximize Growth Performance, Bone Mineralization, and Utilization of Ca and P

In the current study, 3 SID P concentrations were used to determine the SID Ca and SID P requirements in 1- to 10-day-old broilers. As stated earlier, a range of digestible P (4–6 g/kg) which is below and above the recommended dietary P concentration (4.8 g/kg available P; Ross, 2019) were considered in the development of dietary treatments. Based on current data, the SID P concentration of 5 g/kg is recommended for broiler starters (1–10 d post-hatch). The results further indicate that any increase in dietary SID P requires increased dietary SID Ca to maximize both growth performance and bone mineralization. The current finding is in agreement with the SID P values (5.3 and 5.4 g/kg, respectively) reported by Angel (2018) and van Krimpen et al. (2016) for 0 to 10-day-old broilers.

### Requirements for SID Ca to Maximize Growth Performance

In the present study, increasing dietary Ca reduced the feed intake when the dietary SID P was lower (4 g/kg). Consequently, a decline in body weight gain was observed as dietary Ca increased at the dietary SID P of 4 g/kg, suggesting a detrimental effect of Ca on growth performance at low dietary P. Similarly, Walk et al*.* (2012b) reported a reduced feed intake and weight gain with increasing dietary Ca concentrations (4.5, 6.0, 7.5, 9.0 g/kg) in broilers. Similarly, Abdollahi et al. (2016) reported a reduced feed intake and weight gain with increasing dietary Ca concentrations (1.3, 4.3, 7.3, 10.3, and 13.3 g/kg) in broilers. However, the negative effect of Ca at 4 g/kg SID P was ameliorated as the dietary P concentration was increased to 5 and 6 g/kg. The reason for the negative effect on growth performance with increasing dietary Ca concentrations is likely the formation of Ca-P complexes in the digestive tract due to excess Ca and the resultant reduction in P digestibility (Stein et al., 2011; Mutucumarana et al., 2014a). Similar findings of negative Ca effects have been reported in pigs (González-Vega et al., 2016b; Merriman et al., 2017; Lagos et al., 2019a, 2019b) and poultry (Hurwitz et al., 1995; Xie et al., 2009; Akter et al., 2016; Kim et al., 2017).

The linear response of FCR to increasing dietary SID Ca observed in the current study prevented prediction of a maximum response and it was not possible to estimate an optimal concentration of SID Ca at each concentration of SID P for FCR. The FCR data also cannot be used as a reliable parameter to measure the mineral requirement for growth performance because of the wide variation in body weights of broilers in the current study.

For the reasons presented above, the SID P concentration recommended for maximum growth performance of 1 to 10-day-old broilers is 5 g/kg. The concentrations of SID Ca that maximized body weight gain and feed intake at 5 g/kg SID P were 3.32 and 2.33 g/kg, respectively, which correspond to SID Ca to SID P ratios of 0.66 and 0.47, respectively. For feed intake, although the quadratic Ca effect was not significant in the full model, it was included in the model as there was no difference in the R^2^ values between the models with and without quadratic Ca effect. This inclusion of nonsignificant quadratic Ca in the model may have lowered the predicted value of SID Ca (2.33 g/kg) at 5 g/kg for feed intake when compared to that for the weight gain (3.32 g/kg). In addition, it must be noted that a maximum value was not calculated for feed intake at 4 g/kg SID P due to the lack of response. Overall, these predicted values indicate that the growth of broiler starters is maximized below the current Ross (2019) Ca recommendation (9.6 g/kg total Ca or 4.4 g/kg SID Ca).

### Requirements for SID Ca to Maximize Standardized Ileal Digestibility and Intake of Ca and P

The quantity of Ca and P consumed and the ratio between them influence the absorption and post-absorptive utilization of these minerals. However, in the current study, the standardized ileal Ca digestibility was not influenced by the dietary concentrations or ratios of SID Ca or SID P. Similarly, Mutucumarana et al. (2014a) found no effect of increasing dietary Ca concentrations (6, 9, and 12 g/kg) on the ileal Ca digestibility. However, SID Ca intake, a function of feed intake and SID Ca, was linearly increased as the dietary SID Ca concentration increased, which was expected. Similarly, Abdollahi et al. (2016) reported an increased total Ca intake with increasing dietary Ca concentrations (1.3, 4.3, 7.3, 10.3, and 13.3 g/kg) in 1- to 7-day-old broilers. As noted earlier, reduced feed intake with increasing dietary SID Ca further suggests that the birds adjust their feed intake depending on dietary Ca concentration. As a result, it was not possible to estimate an optimal concentration of SID Ca at a given concentration of SID P for these parameters.

Dietary P plays a vital role in skeletal development and synthesis of other tissues in broilers. Absorption and retention of P in broilers is affected by multitude of interacting factors including dietary concentrations of Ca (Mutucumarana et al., 2014a) and P (Rodehutscord et al., 2012), phytic acid (Ravindran et al., 2006), phytase (Selle et al., 2009; David et al., 2020b), and age of birds (Fonolla et al. 1981). In the current study, the coefficient of standardized ileal P digestibility was increased with decreasing dietary Ca and with increasing SID P concentrations, confirming the negative effect of excess Ca for ileal P absorption. Similarly, the ileal P digestibility has been shown to increase with decreasing dietary Ca concentration (Mutucumarana et al., 2014a) and increasing SID P concentrations (Rodehutscord et al., 2012) in broilers. The finding also demonstrates that the standardized ileal P digestibility reduces when the SID Ca to SID P ratio increases, confirming improvements in P digestibility with narrowing Ca:P ratios (Liu et al., 2013; Sebastian et al., 1996; Qian et al., 1996, 1997; Selle et al., 2009). A similar trend was observed in the current study for the SID P intake where the intake was increased with the decreasing dietary Ca concentrations and with increasing SID P concentrations. Based on the response surface model, the concentration of SID Ca that maximized digestible P intake at different SID P concentrations was similar (2.91 g/kg) because there was no interaction between SID Ca and SID P. Corresponding SID Ca to SID P ratio at 5 g/kg SID P was 0.58 which is closer to the ratio (0.66) that maximized the body weight gain of broilers. It must be noted that the digestible P intake was positively correlated (r = 0.67; *P* < 0.001) with the body weight gain in the current study. Dietary P is important for body protein deposition and consequent muscle growth in animals (Bertram, 1995).

### Requirements for Digestible Ca to Maximize Bone Mineralization

Bone mineralization is a process of deposition of minerals (Ca and P) on the organic bone matrix for the development of bones. Almost 99% of ingested Ca and 80% of P is deposited in the bones (Veum, 2010). Concentrations of bone ash and bone Ca and P are 2 criteria that are currently being used to measure the bone mineralization in broilers. In the current study, the bone parameters were influenced by the interaction effect between SID Ca and SID P. As expected, the lowest concentrations of P (4 g/kg) and Ca (3.3 g/kg) reduced the concentrations of ash and Ca and P of tibia. The combination of lowest SID P (4 g/kg) with higher SID Ca resulted in lower concentrations of ash, Ca, and P of tibia compared to all other combinations of SID P and SID Ca. These findings are in agreement with those of Li et al. (2012) who reported lowest concentrations of ash, Ca, and P of tibia in broilers fed a lower non-phytate P (2.7 vs. 5.0 g/kg) and higher total Ca (11.0 vs. 6.0 g/kg) compared to those fed lower concentrations of both non-phytate P and total Ca. Similarly, Rousseau et al. (2013) reported a lower tibia ash in broilers fed a diet with lower non-phytate P of 3.0 g/kg and higher total Ca (10.0 g/kg) compared to those fed other combinations of non-phytate P (3.0 and 4.5 g/kg) and total Ca (6.0 and 10.0 g/kg). These data, along with the findings of the current study, highlight the negative effects of excess Ca on absorbable Ca and P through the formation of insoluble Ca-P complex, but the effect is much greater on P than Ca.

In the current study, concentrations of SID Ca that maximized the concentrations of ash, Ca, and P of tibia and toe ash concentration at 5 g/kg SID P were 4.51, 4.72, 4.36, and 4.78 g/kg, respectively, which correspond to SID Ca to SID P ratios of 0.90, 0.94, 0.87, and 0.96, respectively. These values are well above the requirement of SID Ca and the ratio of SID Ca to SID P for growth performance (2.33–3.32 g/kg and 0.47–0.66, respectively), demonstrating that the birds require Ca and P for bone tissue synthesis beyond the needs for body tissues (Gautier et al., 2017). Similar findings have been reported in pigs (González-Vega et al., 2016b; Lagos et al., 2019a,b). A digestible Ca requirement of 6.1 g/kg has been proposed by Angel (2018) for 0 to 10 d old broilers, but a detailed basis for this recommendation was not provided.

Tibia ash is the most frequently used criterion for the determination of Ca and P requirements in poultry. Early studies have used tibia ash as a criterion to determine the biological value of inorganic phosphates (Nelson and Peeler, 1961; Nelson and Walker, 1964). Subsequently, toe ash was also used as an alternative to determine the bioavailability of P in various phosphate sources (Fritz and Roberts, 1968; Potter, 1988; Potter et al., 1995; Ravindran et al., 1995). However, some studies reported that the toe ash was not an appropriate criterion as the tibia ash (Huff, 1980; Scholey and Buron, 2017) while others reported that the toe ash was more sensitive than tibia ash (Ravindran et al., 1995). In the current study, the responses of tibia ash and toe ash to different dietary Ca and P concentrations were similar which agrees with the finding of Potter (1988) and Jiménez-Moreno et al. (2013). The toe ash concentration was positively correlated with those of tibia ash (r = 0.94; *P* < 0.001), tibia Ca (r = 0.83; *P* < 0.001) and tibia P (r = 0.91; *P* < 0.001), suggesting that toe ash can be used as an effective criterion to determine bone mineralization in broilers.

### Requirements for SID Ca to Maximize Total Tract Retention of Ca and P

Retention of dietary Ca is important to build up the skeletal system. Absorption and retention of Ca may be affected by dietary, physiological, and animal factors. In the current study, the total tract Ca retention and retainable Ca contents were influenced by the interaction between Ca and P, suggesting the importance of formulating diets with proper Ca and P ratio. The linear nature of Ca retention with increasing dietary SID Ca prevented the prediction of maximum response and it was not possible to estimate an optimal concentration of SID Ca for Ca retention at different concentrations of SID P. Although Ca digestibility was unaffected by dietary treatments, both percentage (intake to output) and absolute (g/bird) Ca retentions were reduced at lower dietary SID P concentrations (4 g/kg), suggesting the need to maintain appropriate dietary P to maximize the Ca retention. In addition, the Ca retention was similar in birds fed SID P concentrations 5 and 6 g/kg, regardless of dietary Ca concentrations. Interestingly, the retained Ca followed a trend similar to that of the bone Ca deposition in the current study where the bone Ca concentrations were similar at 5 and 6 g/kg SID P concentrations. In addition, at the lowest P concentration (4 g/kg), both Ca retention and bone Ca were higher at 3.3 g/kg SID Ca compared to higher Ca concentrations, highlighting the negative effect of excess Ca on Ca retention (Gautier et al., 2017). These findings further confirm the verity that almost all the Ca is stored in the skeletal tissue (Veum, 2010). It is also worth noting that Ca retention was positively correlated (r = 0.61; *P* < 0.001) with tibia Ca in the current study. However, dietary Ca requirement studies on pigs (González-Vega et al., 2016a,b) found that the SID Ca to SID P ratio needed to maximize Ca retention was higher than the ratio needed to maximize the bone Ca.

In contrast to the trend in ileal P absorption, P retention was influenced by the interaction between SID Ca and SID P where the retention was reduced at the highest dietary P concentration (6 g/kg), indicating urinary excretion of absorbed P. When the dietary P is above the physiological threshold for maximum retention, additional P is known to be excreted through the kidney (Leske and Coon, 2002). At 6 g/kg SID P, the percentage P retention was lower than the percentage P absorption. However, the P retention values were higher than the P absorption values at 4 g/kg SID P. At 5 g/kg SID P, the percentage P retention was comparable to percentage P absorption, indicating that most of the absorbed P was retained at 5 g/kg dietary SID P. These findings further confirm that 5 g/kg SID P is the more appropriate requirement for 1- to 10-day-old broilers. At 5 g/kg SID P, the retained P (g/bird) was lowest at the 5.5 g/kg dietary SID Ca concentration, further confirming the negative effect of excess Ca on P absorption and retention (Mutucumarana et al., 2014a). Based on the response surface model, the linear nature of P retention to increasing dietary SID Ca observed in the current study prevented the prediction of a maximum response. A negative correlation (r = −0.31; *P* < 0.01) was observed between the P retention and tibia P results in the current study was unexpected. Ostensibly most of the retained P was utilized for the growth of broilers as P utilization is highly correlated with muscle protein synthesis (Bertram, 1995). The existence of strong, positive correlation (r = 0.84; *P* < 0.001) between retained P (g/bird) and body weight gain further lends support to this thesis.

In the current study, the ratio between retained SID Ca and retained SID P was positively correlated (r = 0.56; *P* < 0.001) with the ratio between SID Ca intake and SID P intake. Regardless of dietary treatments, the ratio between retained SID Ca and retained SID P ranged from 0.92 to 1.41, with the upper range being at SID Ca concentrations above 5.0. At 5 g/kg dietary SID P, the ratios ranged from 0.98 to 1.41 depending on the dietary SID Ca concentrations. However, the body weight gain was maximized at lower dietary SID Ca concentrations of 3.3 g/kg and the ratio of retained SID Ca to retained SID P at 3.3 g/kg SID Ca and 5 g/kg SID P was 0.99. Corresponding ratio of retained SID Ca to retained SID P for maximum bone mineralization was 1.11 in the current study.

#### Requirements for SID Ca to Maximize Carcass Retention of Ca and P

The Ca concentration of carcasses of day-old and 10-day-old birds in the current study was 12.4 and 8.2 to 19.0 g/kg, respectively. Ostensibly, the use of different dietary Ca and P concentrations resulted in wide range of carcass Ca concentration in 10-day-old broilers in the current study. Caldas et al. (2019) studied carcass composition of broilers at different ages and reported a carcass Ca concentration of 11.7 g/kg in day-old birds and a Ca concentration of 18.9 g/kg at 12 d of age in birds fed 9.0 g/kg dietary Ca which are comparable to the values in the current work. The carcass P concentration of day-old and 10-day-old birds in the current study were 10.8 and 10.0 to 15.6 g/kg, respectively. Similarly, Caldas et al. (2019) reported a carcass P concentration of 10.3 g/kg in day-old chicks and 15.3 g/kg in 12-day-old broilers.

A notable observation was that the carcass Ca and P retention was representative of total tract retention values. The retained (g/bird) carcass Ca (r = 0.66; *P* < 0.001) and P (r = 0.60; *P* < 0.001) was positively correlated with the total tract Ca and P retention (g/bird), giving additional strength to the determined total tract retention estimates. It must be noted that the total excreta samples were collected during all 10 d of the experimental period. Interestingly, at 5 g/kg SID P, the carcass Ca retention and the tibia Ca were maximized at a SID Ca concentration of 4.72 to 4.74 whereas the carcass P retention and tibia P were maximized at a SID Ca concentration of 4.35 to 4.36, suggesting a close association between the tibia mineralization and the carcass mineral retention. Such an association is to be expected since 990 g/kg of Ca in the body is in the bones.

In conclusion, the present data demonstrated that growth performance, bone mineralization, and Ca and P utilization of broiler starters were optimized at 5 g/kg SID P concentration. The estimate of 5.0 g/kg SID P compares closely to the current Ross (2019) recommendation for available P (4.8 g/kg). Growth performance was negatively affected by dietary SID Ca concentrations above 5 g/kg at the SID P concentration of 5 g/kg or below. The SID Ca required for maximum weight gain and bone mineralization is 3.32 and 4.36 to 4.78, respectively, at 5 g/kg SID P, which correspond to SID Ca to SID P ratios of 0.66 and 0.87 to 0.96, respectively. The current Ross (2019) Ca recommendation (9.6 g/kg total Ca or 4.4 g/kg SID Ca) for broiler starters is higher than the current estimate (3.32 g/kg SID Ca) for weight gain. However, the bone mineralization is maximized around the current total Ca requirement at 8.9 to 9.8 g/kg (4.36–4.78 g/kg SID Ca). Bone mineralization requires more Ca than growth performance demonstrating, as expected, that the birds use Ca exclusively for the synthesis of bone tissues.

**Table 5:** Comparison of calculated and determined^1^ values of standardized ileal digestible calcium (SID Ca) and standardized ileal digestible phosphorous (SID P) of experimental diets (g/kg, as fed basis).

SID Ca	3.3			3.9			4.4			5.0			5.5		
SID P	4.0	5.0	6.0	4.0	5.0	6.0	4.0	5.0	6.0	4.0	5.0	6.0	4.0	5.0	6.0
SID Ca: SID P	0.84	0.67	0.56	0.97	0.78	0.65	1.11	0.89	0.74	1.25	1.00	0.83	1.39	1.11	0.92
Determined SID Ca	3.3	3.3	3.6	3.9	3.7	3.8	4.5	4.3	4.0	5.8	5.1	4.9	5.4	4.9	5.1
Determined SID P	3.4	4.4	6.0	3.3	4.4	5.8	3.3	4.4	5.5	3.2	4.1	5.5	2.9	3.8	5.2
Determined SID Ca: SID P	0.97	0.75	0.60	1.18	0.84	0.66	1.36	0.98	0.73	1.81	1.24	0.89	1.86	1.29	0.98
Difference (calculated minus determined)															
SID Ca	0.0	0.0	−0.3	0.0	0.2	0.1	−0.1	0.1	0.4	−0.8	−0.1	0.1	0.1	0.6	0.4
SID P	0.6	0.6	0.0	0.7	0.6	0.2	0.7	0.6	0.5	0.8	0.9	0.5	1.1	1.2	0.8

1 Dietary Ca or P concentration × Determined SID Ca or SID P for the respective experimental diet.

**Table 6:** Growth performance of broiler chickens fed diets containing different concentrations of standardized ileal digestible (SID) calcium (Ca) and SID phosphorous (P) from d 0 to 10.^1^

SID Ca (g/kg)	SID P (g/kg)	Body weight gain (g/bird)	Feed intake (g/bird)	Feed conversion ratio
3.3	4	240^ef^	258bcd	1.08^a^
	5	257^a^	269^a^	1.06abc
	6	241def	255^cd^	1.06^ab^
3.9	4	240^ef^	254cde	1.05abc
	5	253^ab^	268^ab^	1.07^a^
	6	242def	256^cd^	1.06abc
4.4	4	224^g^	240^f^	1.07^a^
	5	251abc	260abc	1.04bcd
	6	248bcd	258bcd	1.04bcd
5.0	4	213^h^	225^g^	1.07^a^
	5	244cde	253cde	1.04^cd^
	6	248bcd	252cde	1.02^d^
5.5	4	204^i^	220^g^	1.08^a^
	5	235^f^	245^ef^	1.06abc
	6	244cde	250def	1.02^d^
SEM^2^		2.8	3.6	0.009
Main effects				
SID Ca				
3.3		246	261	1.07
3.9		245	259	1.06
4.4		241	253	1.05
5.0		235	243	1.04
5.5		228	238	1.05
SEM		1.6	2.1	0.005
SID P				
4		224	239	1.07
5		248	259	1.05
6		245	254	1.04
SEM		1.3	1.6	0.004
Probabilities, *P* ≤				
SID Ca		< 0.001	< 0.001	0.008
SID P		< 0.001	< 0.001	< 0.001
SID Ca × SID P		< 0.001	< 0.001	0.032

a-i Means having different superscripts within the column are significantly different (*P* < 0.05).

1 Each value represents the mean of 6 replicates (12 birds per replicate).

2 Pooled standard error of mean.

**Table 7:** Standardized ileal digestibility coefficient (SIDC) of calcium (Ca) and phosphorous (P), intake (g/bird) of standardized ileal digestible (SID) Ca and SID P, and the ratio of SID Ca intake to SID P intake, in broiler chickens fed different concentrations (g/kg) of SID Ca and SID P from d 0 to 10.^1^

SID Ca	SID P	SIDC of Ca	SID Ca intake	SIDC of P	SID P intake	SID Ca intake: SID P intake
3.3	4	0.47	0.85	0.56	0.87	0.98^d^
	5	0.46	0.88	0.60	1.18	0.74^fg^
	6	0.51	0.92	0.69	1.54	0.60^h^
3.9	4	0.50	0.99	0.54	0.83	1.20^c^
	5	0.47	0.98	0.60	1.17	0.84^ef^
	6	0.48	0.97	0.68	1.49	0.65^gh^
4.4	4	0.51	1.09	0.54	0.79	1.39^b^
	5	0.49	1.13	0.60	1.15	0.97^d^
	6	0.46	1.04	0.64	1.42	0.73^fg^
5.0	4	0.54	1.31	0.52	0.71	1.83^a^
	5	0.48	1.29	0.56	1.03	1.25^c^
	6	0.46	1.24	0.63	1.39	0.89^de^
5.5	4	0.48	1.19	0.48	0.64	1.85^a^
	5	0.43	1.21	0.52	0.93	1.29^bc^
	6	0.45	1.28	0.60	1.30	0.98^d^
SEM^2^		0.024	0.058	0.015	0.031	0.043
Main effects						
SID Ca						
3.3		0.48	0.89^d^	0.62^a^	1.20^a^	0.77
3.9		0.49	0.98^c^	0.61^a^	1.16^ab^	0.90
4.4		0.49	1.08^b^	0.59^ab^	1.12^b^	1.03
5.0		0.49	1.28^a^	0.57^b^	1.04^c^	1.32
5.5		0.45	1.22^a^	0.53^c^	0.96^d^	1.38
SEM		0.014	0.034	0.009	0.018	0.025
SID P						
4		0.50	1.09	0.53^c^	0.77^c^	1.45
5		0.47	1.10	0.57^b^	1.09^b^	1.02
6		0.47	1.09	0.65^a^	1.43^a^	0.77
SEM		0.011	0.026	0.007	0.014	0.019
Probabilities, *P* ≤						
SID Ca		0.301	< 0.001	< 0.001	< 0.001	< 0.001
SID P		0.071	0.938	< 0.001	< 0.001	< 0.001
SID Ca × SID P		0.391	0.878	0.664	0.848	< 0.001

a-h Means having different superscripts within the column are significantly different (*P* < 0.05).

1 Each value represents the mean of 6 replicates (12 birds per replicate).

2 Pooled standard error of mean.

**Table 8:** Concentration of ash, calcium (Ca) and phosphorous (P) in tibia (g/kg dried defatted matter) and toe ash concentration (g/kg, as received basis) in broiler chickens fed diets containing different concentrations (g/kg) of standardized ileal digestible (SID) Ca and SID P from d 0 to 10.^1^

SID Ca	SID P	Tibia ash	Tibia Ca	Tibia P	Toe ash
3.3	4	354^de^	115^de^	56.0^c^	34.8^de^
	5	395^bc^	126^bc^	64.3^ab^	40.3^c^
	6	384^c^	121^cd^	62.5^b^	40.8^c^
3.9	4	357^d^	116^de^	56.0^c^	35.7^d^
	5	401^ab^	129^ab^	65.1^ab^	40.9^c^
	6	401^ab^	127^ab^	65.8^ab^	44.3^a^
4.4	4	341^ef^	109^ef^	53.1^cd^	33.1^f^
	5	409^a^	133^ab^	66.3^a^	42.5^b^
	6	408^ab^	132^ab^	66.7^a^	44.5^a^
5.0	4	336^fg^	110^ef^	51.7^de^	33.4^ef^
	5	411^a^	131^ab^	66.3^a^	44.9^a^
	6	411^a^	135^a^	67.3^a^	44.4^a^
5.5	4	322^g^	102^f^	49.4^e^	31.6^g^
	5	410^a^	134^ab^	66.6^a^	44.4^a^
	6	411^a^	135^ab^	66.6^a^	44.4^a^
SEM^2^		5.0	3.1	1.24	0.54
Main effects					
SID Ca					
3.3		377	121	60.9	38.6
3.9		387	124	62.3	40.3
4.4		386	125	62.0	40.0
5.0		386	125	61.8	40.9
5.5		381	124	60.9	40.1
SEM		2.9	1.8	0.71	0.31
SID P					
4		342	110	53.3	33.7
5		405	131	65.7	42.6
6		403	130	65.8	43.7
SEM		2.2	1.4	0.55	0.24
Probabilities, *P* ≤					
SID Ca		0.108	0.458	0.513	< 0.001
SID P		< 0.001	< 0.001	< 0.001	< 0.001
SID Ca × SID P		< 0.001	0.001	0.001	< 0.001

a-g Means having different superscripts within the column are significantly different (*P* < 0.05).

1 Each value represents the mean of 6 replicates (8 birds per replicate).

2 Pooled standard error of means.

**Table 9:** Apparent total tract retention coefficients (ATTRC) of calcium (Ca) and phosphorous (P) and retained Ca and P in 10-day-old broilers fed diets containing different concentrations of (g/kg) standardized ileal digestible (SID) Ca and SID phosphorous (P).^1^

SID Ca (g/kg)	SID P (g/kg)	ATTRC of Ca	Retained Ca (g/bird)	ATTRC of P	Retained P (g/bird)	Retained Ca: retained P ratio
3.3	4	0.51^cd^	0.93^gh^	0.65^a^	1.02^f^	0.92^e^
	5	0.60^a^	1.14^de^	0.58^bc^	1.15bcd	0.99^e^
	6	0.57^ab^	1.03^fg^	0.49^e^	1.08def	0.96^e^
3.9	4	0.47^e^	0.93^gh^	0.60^bc^	0.93^g^	1.01^e^
	5	0.55^bc^	1.14^de^	0.60^bc^	1.17^ab^	0.98^e^
	6	0.57^ab^	1.13^de^	0.51^e^	1.12bcde	1.01^e^
4.4	4	0.40^f^	0.84^h^	0.61^b^	0.88^g^	0.96^e^
	5	0.55^bc^	1.28^bc^	0.60^bc^	1.15^bc^	1.11^d^
	6	0.53^bc^	1.21^cd^	0.55^d^	1.23^a^	0.99^e^
5.0	4	0.46^e^	1.10^ef^	0.59^bc^	0.80^h^	1.37^ab^
	5	0.57^ab^	1.53^a^	0.58^bc^	1.08cdef	1.41^a^
	6	0.54^bc^	1.47^a^	0.51^e^	1.13bcde	1.29^bc^
5.5	4	0.40^f^	1.01^fg^	0.57^cd^	0.77^h^	1.32^ab^
	5	0.53^c^	1.46^a^	0.59^bc^	1.06^ef^	1.38^ab^
	6	0.48^cd^	1.35^b^	0.51^e^	1.12bcde	1.21^c^
SEM^2^		0.014	0.036	0.011	0.026	0.033
Main effects						
SID Ca						
3.3		0.56	1.03	0.57	1.08	0.96
3.9		0.53	1.07	0.57	1.07	1.00
4.4		0.49	1.11	0.59	1.09	1.02
5.0		0.52	1.36	0.56	1.00	1.36
5.5		0.47	1.27	0.56	0.98	1.30
SEM		0.008	0.021	0.006	0.015	0.019
SID P						
4		0.45	0.96	0.60	0.88	1.12
5		0.56	1.31	0.59	1.12	1.17
6		0.54	1.24	0.51	1.13	1.09
SEM		0.006	0.016	0.005	0.012	0.015
Probabilities, *P* ≤						
SID Ca		< 0.001	< 0.001	0.027	< 0.001	< 0.001
SID P		< 0.001	< 0.001	< 0.001	< 0.001	< 0.001
SID Ca × SID P		0.042	< 0.001	< 0.001	< 0.001	0.037

a-h Means having different superscripts within the column are significantly different (*P* < 0.05).

1 Each value represents the mean of 6 replicates (8 birds per replicate).

2 Pooled standard error of mean.

**Table 10:** Retention (g/bird) of calcium (Ca) and phosphorous (P) in the carcass of 10-d old broilers fed diets containing different concentrations of (g/kg) standardized ileal digestible (SID) Ca and SID P.^1^^,^^2^^,^^3^

SID Ca	SID P	Ca	P
3.3	4	0.94^f^	0.98^de^
	5	1.14^de^	1.14^bc^
	6	1.09^ef^	1.08^cd^
3.9	4	0.93^fg^	0.94^e^
	5	1.26cde	1.17abc
	6	1.27bcd	1.16^bc^
4.4	4	0.94^fg^	0.91^e^
	5	1.41abc	1.24^ab^
	6	1.38abc	1.23^ab^
5.0	4	0.77^gh^	0.79^f^
	5	1.38abc	1.20^ab^
	6	1.46^a^	1.26^a^
5.5	4	0.74^h^	0.75^f^
	5	1.44^ab^	1.21^ab^
	6	1.38abc	1.22^ab^
SEM^3^		0.063	0.037
Main effects			
SID Ca			
3.3		1.06	1.07
3.9		1.15	1.09
4.4		1.24	1.13
5.0		1.20	1.08
5.5		1.18	1.06
SEM		0.036	0.021
SID P			
4		0.86	0.88
5		1.33	1.19
6		1.32	1.19
SEM		0.028	0.016
			
Probabilities, *P* ≤			
SID Ca		0.001	0.198
SID P		0.001	0.001
SID Ca × SID P		0.009	0.001

a-e Means having different superscripts within the column are significantly different (*P* < 0.05).

1 Each value represents the mean of 6 replicates (4 birds per replicate). The term ‘carcass’ refers to the whole body without feathers.

2 Ca and P in the carcass of day-old bird is 0.140 and 0.123 g/bird, respectively, and these values are deducted from the total retained Ca or P at d 10.

3 Pooled standard error of mean.

**Table 11:** Analyzed nutrient and mineral composition of calcium and phosphorous (P) supplements (g/kg, as received basis).^1^

Nutrient	Corn	Soybean meal	Limestone	Dicalcium phosphate	Monosodium phosphate
Dry matter	897	906	1,000	969	974
Ash	12	64	996	848	828
Crude protein	75	456	-	-	-
Fat	37	25	-	-	1.0
Neutral detergent fiber	85	82	-	-	-
Calcium	0.2	3.5	400	260	-
Total P	2.3	5.9	0.56	190	225
Phytate	5.0	11	-	-	-
Phytate P	1.5	3.0	-	-	-
Non-phytate P^2^	0.8	2.9	-	-	-
Sodium	< 0.05	< 0.05	< 0.50	0.71	196

1 Samples were analyzed in duplicate.

2 Calculated as the difference between total P and phytate P.

## DISCLOSURES

No conflict of interest is declared.

## Appendix Supplementary materials

Image, application 1
