# Supplementary material for: Requirement of digestible calcium at different dietary concentrations of digestible phosphorus for broiler chickens. 1. Broiler starters (d 1 to 10 post-hatch)
Source: Poult Sci. 2021 Aug 19;100(11):101439. doi: 10.1016/j.psj.2021.101439 (PMC8493592; doi:10.1016/j.psj.2021.101439)
Supplement: Supplementary file 1 [file mmc1.docx]

## Supplementary data

## Response surface models

| a)  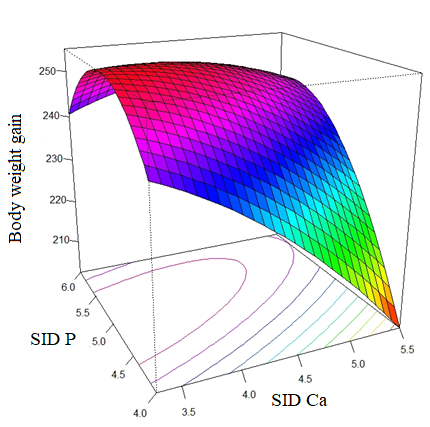 | b)  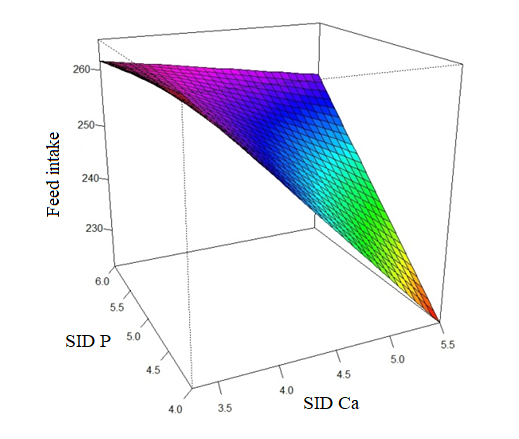 |
| --- | --- |
| c)  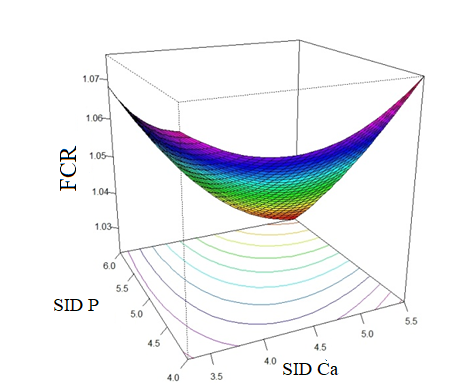 | |

Figure 1. (a) Body weight gain (g/bird), (b) feed intake (g/bird) and (c) feed conversion ratio (FCR) of broiler chickens fed different concentrations (g/kg) of standardised ileal digestible (SID) calcium (Ca) and SID phosphorous (P) from day 1 to 10.

| 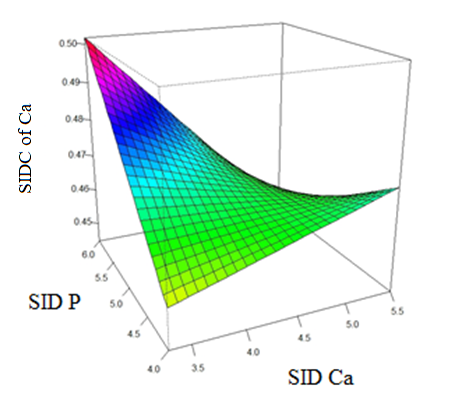a) | 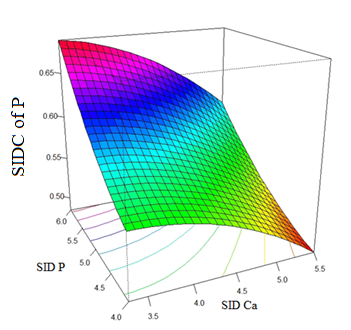b) |
| --- | --- |
| c)  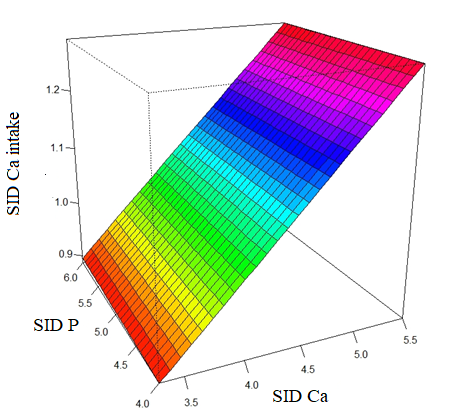 | d)  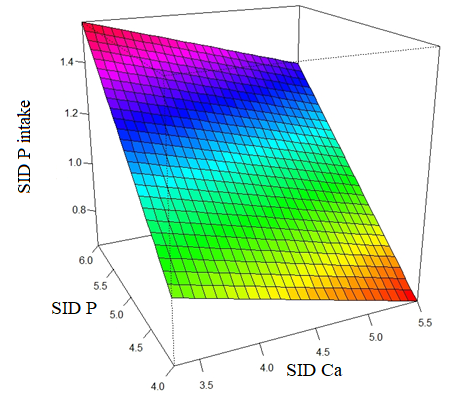 |
| 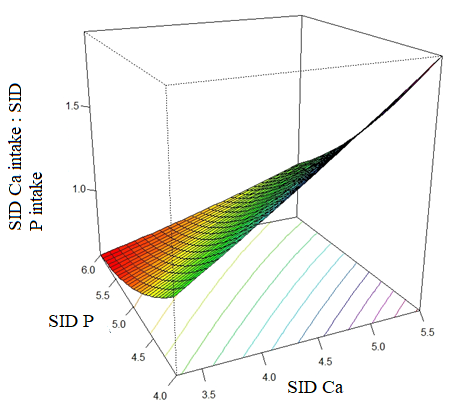e) | |

Figure 2. Standardised ileal digestibility coefficient (SIDC) of a) calcium (Ca) and b) phosphorous (P), Intakes (g/bird) of c) standardised ileal digestible (SID) Ca and b) SID P, and c) ratio of SID Ca intake to SID P intake, of broiler chickens fed different concentrations (g/kg) of SID Ca and SID P from day 1 to 10

| a)  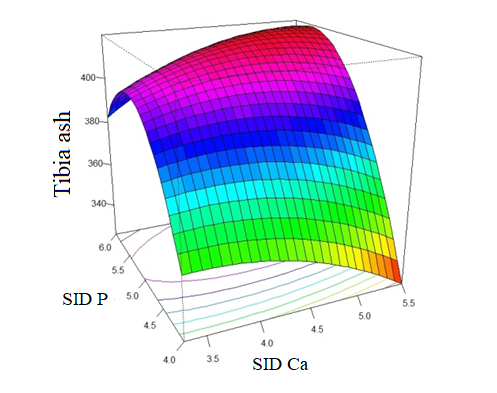 | b)  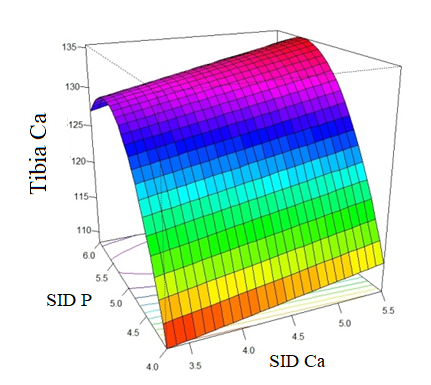 |
| --- | --- |
| c)  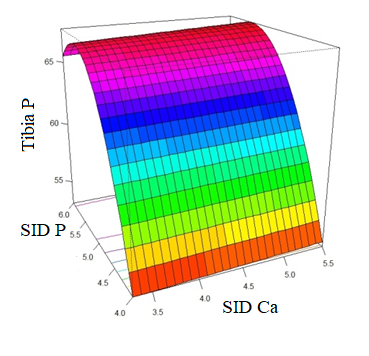 | d)  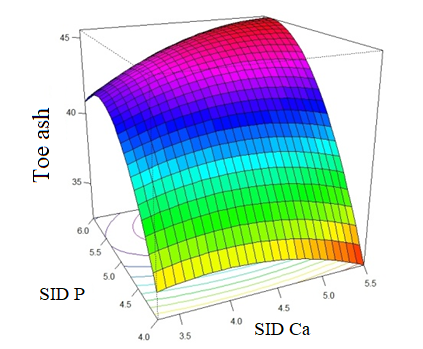 |

Figure 3. Concentrations (g/kg dried defatted matter) of a) ash, b) calcium (Ca) and c) phosphorous (P) of tibia and d) toe ash content (g/kg, as received basis) in broiler chickens fed different concentrations (g/kg) of standardised ileal digestible (SID) Ca and SID P from day 1 to 10

| a)  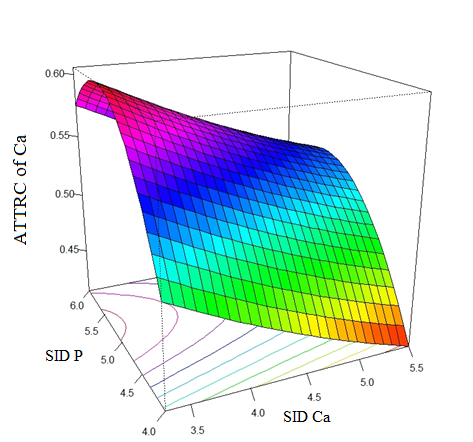 | b)  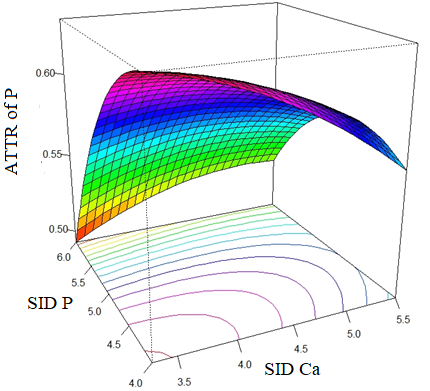 |
| --- | --- |
| c)  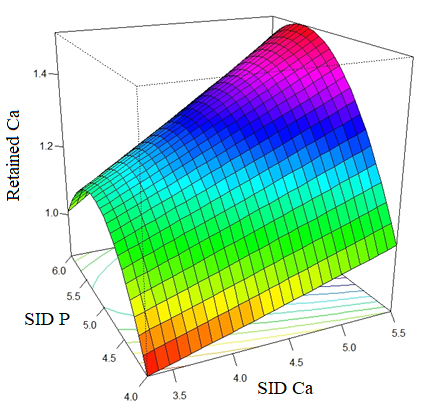 | d)  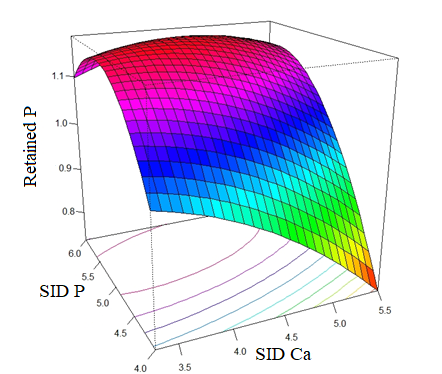 |
| e)  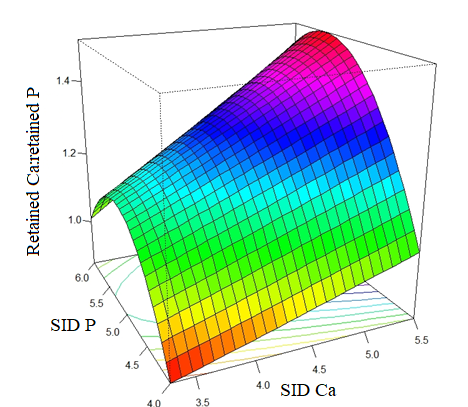 | |

Figure 4. a) Apparent total tract retention coefficient (ATTRC) of calcium (Ca), b) ATTRC of phosphorous (P), c) total tract retained Ca (g/bird), d) total tract retained P (g/bird), e) ratio of retained Ca to retained P, of broiler chickens fed different concentrations (g/kg) of standardised ileal digestible (SID) Ca and SID P from day 1 to 10

| 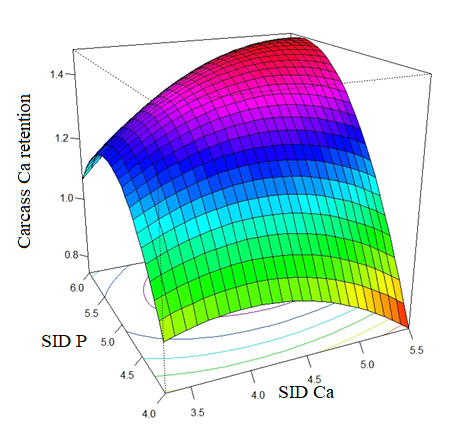a) | 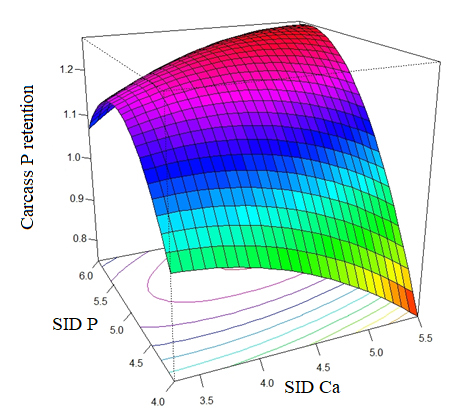b) |
| --- | --- |

Figure 5. Carcass retention (g/bird) of a) calcium (Ca) and b) phosphorous (P) of broiler chickens fed different concentrations (g/kg) of standardised ileal digestible (SID) Ca and SID P from day 1 to 10
